# Digital quantum simulation of NMR experiments

**DOI:** 10.1126/sciadv.adh2594

**Published:** 2023-11-17

**Authors:** Kushal Seetharam, Debopriyo Biswas, Crystal Noel, Andrew Risinger, Daiwei Zhu, Or Katz, Sambuddha Chattopadhyay, Marko Cetina, Christopher Monroe, Eugene Demler, Dries Sels

**Affiliations:** ^1^ Department of Electrical Engineering, Massachusetts Institute of Technology, Cambridge, MA 02139, USA.; ^2^ Department of Physics, Harvard University, Cambridge, MA 02138, USA.; ^3^ Department of Electrical and Computer Engineering, Department of Physics, Duke Quantum Center, Duke University, Durham, NC 27708, USA.; ^4^ Joint Quantum Institute, Department of Physics, University of Maryland, College Park, MD 20742, USA.; ^5^ Department of Physics, Duke Quantum Center, Duke University, Durham, NC 27708, USA.; ^6^ IonQ Inc., College Park, MD 20740, USA.; ^7^ Institute for Theoretical Physics, ETH Zürich, 8093 Zürich, Switzerland.; ^8^ Department of Physics, New York University, New York, NY 10003, USA.; ^9^ Center for Computational Quantum Physics, Flatiron Institute, New York, NY 10010, USA.

## Abstract

Simulations of nuclear magnetic resonance (NMR) experiments can be an important tool for extracting information about molecular structure and optimizing experimental protocols but are often intractable on classical computers for large molecules such as proteins and for protocols such as zero-field NMR. We demonstrate the first quantum simulation of an NMR spectrum, computing the zero-field spectrum of the methyl group of acetonitrile using four qubits of a trapped-ion quantum computer. We reduce the sampling cost of the quantum simulation by an order of magnitude using compressed sensing techniques. We show how the intrinsic decoherence of NMR systems may enable the zero-field simulation of classically hard molecules on relatively near-term quantum hardware and discuss how the experimentally demonstrated quantum algorithm can be used to efficiently simulate scientifically and technologically relevant solid-state NMR experiments on more mature devices. Our work opens a practical application for quantum computation.

## Teaser

A quantitatively accurate simulation of an organic compound NMR experiment is demonstrated on a trapped-ion quantum computer.

## INTRODUCTION

Nuclear magnetic resonance (NMR) spectroscopy is a widely used tool in structural biology and materials chemistry, providing insight into the structure, dynamics, reaction state, and chemical environment of both liquid-state and solid-state systems (*1*). Systems studied with liquid-state NMR range from to small biomolecules such as metabolites to large biomacromolecules such as proteins, nucleic acids, carbohydrates, and lipids, while solid-state NMR can help characterize various rigid and semi-rigid systems such as membrane proteins, amyloid fibrils, polymers, battery materials, photovoltaic perovskites, solid state catalysts, and metal-organic frameworks (*2*, *3*). Despite their versatility, NMR experiments can be difficult to interpret for large liquid- and solid-state systems. Current approaches typically combine sophisticated experimental protocols that simplify the nuclear spin dynamics at the heart of the experiment with heuristic formulas to infer the information of interest. When available, numerical simulation of the spin dynamics can provide an important tool in the analysis and optimization of conventional high-field liquid-state experiments on large biomolecules (*4*), emerging zero-field experiments on small biomolecules (*5*, *6*), and many high-field solid-state experiments (*2*). For example, simulation can be used to optimize experimental protocols and pulse sequences, validate chemical and structural information extracted from heuristic formulas applied to the experiment, and open the door to novel structure determination paradigms (*4*).

Numerical simulations of NMR experiments, however, can be very challenging to perform on classical computers when either the dynamics itself is hard, meaning that correlations generated by the quantum dynamics of the system becomes difficult to keep track of (see the Supplementary Materials), or when an ensemble (“powder”) average over system orientations is performed (*2*). Liquid-state NMR exhibits complex dynamics in zero-field experiments dominated by coherent exchange interactions even for relatively small biomolecules and in high-field experiments dominated by dipolar relaxation for large biomacromolecules. Solid-state NMR, dominated by coherent dipolar interactions, often exhibits hard dynamics and necessitates powder averaging over differently oriented grains in the sample.

Quantum computers and simulators, themselves described as an effective system of spins, are naturally suited to simulate the dynamics of spin systems. These simulations may be the first practical application of quantum computers to achieve a speed-up compared to classical computers (*7*). Quantum hardware may therefore enable the simulation of NMR experiments with complex dynamics (*8*). These spin dynamics simulations can enable inference of Hamiltonian parameters encoding the chemical and structural information of interest in NMR experiments. Liquid-state zero-field NMR experiments on relatively small molecules may be the earliest achievable context where quantum computers can display an advantage over classical computers. Solid-state NMR, on the other hand, is a more widely relevant context where quantum computers may have greater practical utility but would require substantial improvements to current quantum hardware to simulate the large systems typically investigated in experiments.

Here, we simulate a zero-field NMR experiment on a trapped-ion quantum computer (*9*). The quantum computer implements a carefully compiled sequence of unitary rotations and entangling interactions on ^171^Yb^+^ ion qubits to implement a digital quantum circuit that emulates the NMR experiment (see the Supplementary Materials). Specifically, we compute the spectrum of selectively isotope-labeled acetonitrile, with four NMR-active nuclear spins, and show that the resonance frequencies in the spectrum quantitatively match the experimental NMR data from (*10*), while the peak intensities match in their ordering.

We obtain high spectral resolution within the resource limitations of the trapped-ion device by exploiting compressed sensing techniques (*11*) and a state-of-the-art quantum circuit synthesis algorithm (*12*). These techniques, which leverage the power of the individual qubit control of a digital quantum computer, enable more versatile simulations of NMR spectra than would be possible on typical analog quantum simulators that seek to natively implement the desired dynamics (*13*). They reduce the resource cost for simulating classically hard NMR systems substantially and are likely to prove useful in quantum simulations of hard systems that appear in quantum chemistry and condensed matter physics (*14*). We give resource estimates for quantum simulations of relatively small molecules whose zero-field NMR spectra are practically challenging to simulate on classical computers, showing how the dephasing commonly present in nuclear spin dynamics may enable these simulations on relatively near-term quantum devices. We then discuss how more mature quantum hardware may enable efficient analysis of large systems such as membrane proteins and battery materials studied using solid-state NMR experiments.

## RESULTS

An NMR experiment involves polarizing the nuclear spins of a sample via an external magnetic field or a chemical process, letting the spins evolve in time, and measuring the average magnetization of the system. The measured time-dependent magnetization is called the free induction decay (FID), and its Fourier transform yields the NMR spectrum. Letting the operators {**S***_i_*} represents the nuclear spins, the initial state of the system when polarized via either a large magnetic field or a chemical process can be described as $\rho_{0}\approx I+\lambda {\tilde{S}}_{\mathrm{tot}}^{z}$, where *I* is the identity operator and ${\tilde{S}}_{\mathrm{tot}}^{z}=\sum_{i}\gamma_{i}S_{i}^{z}$, with γ*_i_* being the gyromagnetic ratio of the nuclear isotope *i*. In the case of a one-dimensional NMR experiment, the measured FID corresponds to the quantity

$$\mathrm{FID}(t)=\mathrm{Tr}[U{\left(t\right)}^{†}{\tilde{S}}_{\mathrm{tot}}^{z}U(t){\tilde{S}}_{\mathrm{tot}}^{z}]$$ (1)

where *U*(*t*) = exp(−*iHt*/ℏ) produces the time evolution of the system generated by a Hamiltonian *H*. Most NMR experiments can be modeled by Eq. 1 with different Hamiltonians and, in the case of multidimensional protocols, global spin rotations that modify the time evolution (*1*). The evolution of liquid-state molecular samples is typically well captured by

$$H=\sum_{i,j}2\pi J_{ij}S_{i}\cdot S_{j}+\sum_{i}\omega_{i}S_{i}^{x}$$ (2)

where we have taken Planck’s constant ℏ = 1. The *J* couplings {*J_ij_*} characterize the strength of bond-mediated exchange interactions, and the chemical shifts {ω*_i_*} represent local magnetic screening around nuclei in different chemical environments in response to an external magnetic field (*1*).

Zero-field NMR protocols avoid the external field, opening the possibility of portable and cheaper experiments as they obviate the need for cryogenically cooled superconducting magnets (*5*, *6*, *10*). Without a large background field, however, the interactions between spins become dominant. Therefore, a notable limitation of zero-field protocols is that their spectra are hard to interpret without access to simulations of the NMR experiment, which can be rendered classically intractable for even relatively small molecules (*15*). High-field liquid-state protocols, in contrast, are typically easy to simulate as the scalar *S_i_* · *S_j_* interaction in Eq. 2 reduces to its secular component $S_{i}^{z}S_{j}^{z}$, which substantially simplifies the complexity of the dynamics.

**Fig. 1.**
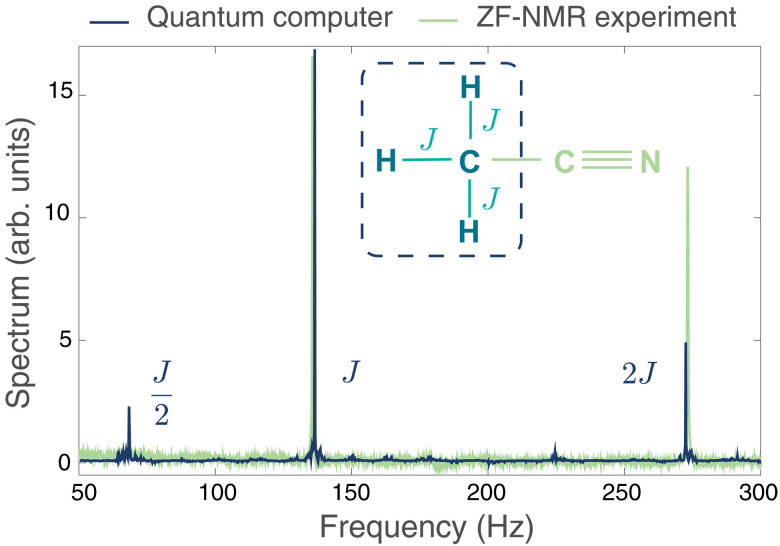
Liquid-state NMR spectrum computed on quantum hardware. Zero-field (ZF) spectrum of acetonitrile computed on an trapped-ion quantum computer (blue curve) compared with the NMR experiment (green curve) performed in (*10*). The inset shows the chemical structure of acetonitrile, highlighting the methyl group that was probed in the experiment. arb. units, arbitrary units.

We compute the zero-field spectrum of acetonitrile, a compound that is commonly used as an industrial solvent. The molecule is isotope-labeled to have four NMR-active spin-1/2 nuclei, a ^13^C and three ^1^H, that make up a methyl group (see inset in Fig. 1). There are three nonzero *J* couplings, corresponding to the three ^13^C─^1^H bonds, all with value *J* = 136.2 Hz. The FID signal of Eq. 1 can be computed on a quantum computer using four qubits by initializing the system qubits in basis states with a positive average magnetization, enacting time evolution under the Hamiltonian via an appropriate quantum circuit (Eq. 2 and then measuring the average magnetization of the system. We write this measurable as

$$\mathrm{FID}(t)=\sum_{{\tilde{m}}_{n}>0}{\tilde{m}}_{n}{\tilde{m}}_{n}(t)|{\tilde{S}}_{\mathrm{tot}}^{z}|{\tilde{m}}_{n}(t)$$ (3)

where $\left\{{\tilde{m}}_{n};{\tilde{m}}_{n}\right\}$ are the eigenstates and eigenvalues of ${\tilde{S}}_{\mathrm{tot}}^{z}$ and ${\tilde{m}}_{n}(t)=U(t){\tilde{m}}_{n}$. For a system of *N* spins, the sum in Eq. 3 can have a number of terms that scales exponentially with *N*, naively negating the quantum computational advantage. However, the variance of the estimator is much smaller than exponential and can be bounded by *N*^2^ due to the bounded operator norm of the total magnetization being measured; at most, *N*^2^ terms of the sum can thus be used to estimate the FID via either uniform or importance sampling (*8*). The described quantum algorithm can be straightforwardly generalized to simulate a diverse array of NMR experiments, including protocols with different initial conditions, multiple dimensions, solid-state samples, and different isotope labeling. Multidimensional protocols can be simulated by inserting single-qubit rotations into the time evolution of *U*(*t*), and solid-state experiments can be simulated by including a dipolar interaction term in the Hamiltonian of Eq. 2 (see the Supplementary Materials). Isotope labeling is incorporated by the choice of basis states that the qubits are prepared in and by the choice of qubits to measure at the end of the simulation.

Figure 1 shows the spectrum we compute on an trapped-ion quantum computer in comparison with the seminal zero-field NMR experiment of (*10*). We see that the quantum computation accurately reproduces the resonances at frequencies *J* and 2*J*. Specifically, the corresponding resonance frequencies extracted from the quantum simulation are 136.20 ± 0.09 and 272.41 ± 0.09 Hz, which are within 1σ of the exact frequencies of 136.2 and 272.4 Hz. The extracted resonance frequency uncertainty is Fourier limited by the total acquisition time; a Lorentzian fit to the reconstructed peaks results in a width smaller than the frequency grid spacing. We therefore take half the grid spacing as the uncertainty. Given that the zero-field NMR experiment can only resolve the spectral peaks within 0.1 Hz (*10*), we demonstrate that quantum computers can accurately simulate NMR data with a quality comparable to real experiments.

The spectrum computed on the quantum computer has peak intensities that match the ordering of peaks in (*10*) but has a quantitative mismatch with spectral weight transferred from the resonance at 2*J* to an additional resonance at *J*/2 that is not present in the NMR experiment. This additional spectral peak arises from a combination of errors in the quantum computer and the high symmetry of the molecule, which induces dynamical recurrences that are captured by the specific method we use to synthesize the time evolution circuits. These artifacts are unlikely to appear in classically intractable NMR simulations whose large, strongly correlated molecules typically do not exhibit many-body revivals. Furthermore, we provide a simple method to remove artifact peaks in future experiments even for the small, highly symmetric systems where they may occur (see the Supplementary Materials).

To calculate the spectrum, we first compute the FID (Eq. 3) at a nonuniform random sampling of time points lower than the Nyquist rate. We synthesize the time evolution quantum circuits using the numerical optimization algorithm in (*12*) after tailoring it to the gate set and qubit topology of the trapped ion device (see the Supplementary Materials). This numerical synthesis procedure efficiently produces low-depth circuits but is limited to a small number of qubits because it searches of the full space of possibly unitaries. It can, however, be a useful tool when simulating larger systems by combining it with a cluster Trotterization method, as described in the Supplementary Materials.

**Fig. 2.**
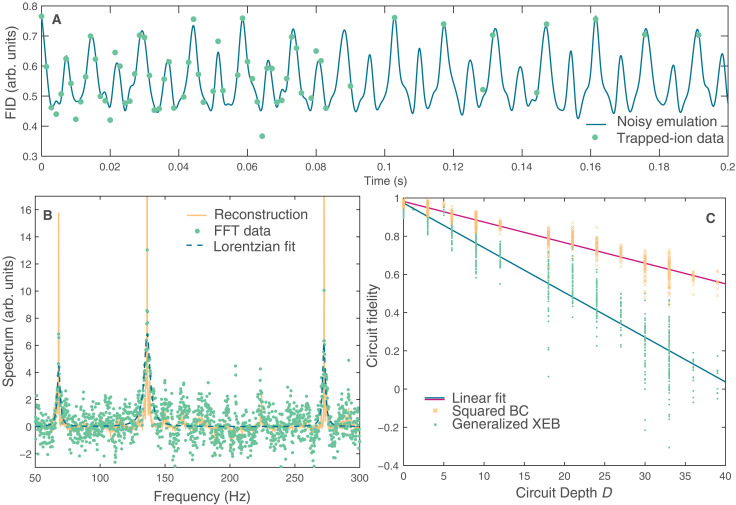
Compressed sensing reconstruction and benchmarking. (**A**) Comparison of the FID of a noisy quantum circuit emulation (blue line) and the nonuniform, sparsely sampled points experimentally measured on the trapped-ion quantum computer (green circles). The noise is modeled by two-qubit gates subject to both amplitude and phase damping with rates of 0.005 and 0.035 s, respectively. (**B**) NMR spectrum extracted from the digital quantum simulation, where the spectrum is the real part of the Fourier transform of the FID. Green dots show the spectrum after replacing unsampled points of the FID with zeros. Dashed blue line shows the best (under 𝓁_1_-norm) Lorentzian fits to this zero-padded data. Solid yellow line shows the reconstructed spectrum after applying the IST-S algorithm. The *y* axis is rescaled (zoomed-in) compared to Fig. 1 to make the features more visible. (**C**) Fidelity of quantum simulation. The yellow crosses show the squared Bhattacharyya coefficient, and the green dots show a generalized cross entropy benchmark (gXEB) (*19*) as a function of the circuit depth measured in the number of two-qubit gates. arb. units, arbitrary units.

The undersampled FID measured in experiment is reconstructed into a spectrum by a recovery algorithm, which assumes that the time domain signal is sparse in the frequency domain. These two steps—nonuniform sampling (NUS) and spectral reconstruction—form the basis of compressed sensing. Compressed sensing techniques have their root in information theory (*16*) but have been further developed in the experimental NMR community where they can markedly reduce the data collection burden (*11*). While these techniques have recently been used in quantum sensing (*17*), we demonstrate their use in quantum simulation experiments to similarly reduce the computational cost (*14*). In Fig. 2A, we plot a noisy emulation of the trapped-ion experiment at all values of the uniform dense time grid and compare to the NUS points that were actually collected in the experiment. Noise is modeled with amplitude and phase damping channels acting on each two-qubit gate, with the rate of each channel determined by fitting the emulation to experimental data. Experimental data were collected up to times *t* = 6 s (see the Supplementary Materials) but are only shown up to *t* = 0.2 s to allow a clear comparison to the noisy emulation. We use a sine-weighted Poisson gap NUS schedule that is dense at short times as it has been shown to reduce reconstruction artifacts (*18*). Figure 2B shows the spectrum resulting from Fourier transforming the experimental data before running the reconstruction algorithm. We see that the signal-to-noise ratio in this raw spectrum is poor because of NUS artifacts, with a Lorentzian fit to the peaks resulting in an uncertainty of approximately 1 Hz. The same spectrum is shown after we run the iterative soft thresholding (IST-S) reconstruction algorithm; the signal-to-noise ratio is markedly improved, with the uncertainty reducing by an order of magnitude to approximately 0.1 Hz. The reconstructed spectrum matches the spectrum resulting from fully sampled noisy emulation (see the Supplementary Materials). Experimentally, only 102 of the 4096 time points were collected, indicating that compressed sensing reduced the computational burden of the experiment by more than a factor of 40. This reduction is particularly crucial for experiments with slow repetition rates. We note that compressed sensing techniques will remain applicable to quantum simulation of NMR experiments on large, classically intractable systems well-beyond the small molecule demonstrated in this work. The multidimensional NMR protocols used to study these systems are designed to spread spectral weight across different dimensions to improve interpretability of the spectrum, thus manifesting the frequency-domain sparsity required for compressed sensing.

In Fig. 2C, we asses the quality of the trapped-ion simulation by comparing the outputs of all 102 circuits (×8 initial states) with the ideal outputs resulting from a noiseless circuit emulation. These synthesized circuits, each corresponding to a time *t*, have varying circuit depths according to the entanglement generated in the system at that time (see the Supplementary Materials). The Bhattacharyya coefficient, which provides an upper bound for the fidelity of the prepared quantum state (see the Supplementary Materials), indicates that a typical two-qubit gate operation was enacted with fidelity at most 98.9%. The Bhattacharyya coefficient is an informative metric for states with high fidelities, but it saturates to a value of 0.5 for the random states that the system tends to after decoherence runs its course. We therefore also examine a generalized cross-entropy benchmark (gXEB) introduced in (*19*), which is a better estimate for the fidelity. The gXEB yields an estimate of 97.7% fidelity per operation enacted in the trapped-ion experiment. It should be noted that, similar to the XEB (*20*), the gXEB can be negative.

While the present experiment is performed on state-of-the-art quantum hardware, it is still easily tractable on a classical computer. To elucidate the hardware resources required to scale quantum simulations to classically hard zero-field NMR experiments, we examine three challenging systems that are at the border of what is classically simulable (*21*, *22*).

**Fig. 3.**
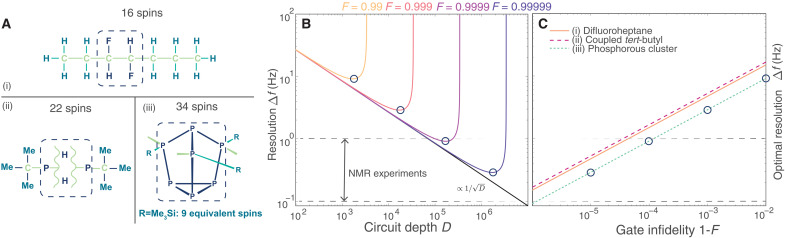
Scaling up to classically hard liquid-state zero-field NMR simulations. (**A**) Chemical structures of (i) anti-3,4-difluoroheptane (*38*), (ii) a system with two coupled *tert*-butyl groups, and (iii) the B[ACR9]3 phosphorous system (*21*). Light green atoms do not contribute to the NMR signal, and dashed boxes indicate strongly interacting clusters where circuit synthesis can substantially speed up the quantum computation (see the Supplementary Materials). (**B**) Experimental design curves for (Me_3_Si)_3_P_7_ [(A), iii], showing $1/\sqrt{D}$ scaling, where *D* is the circuit depth, of the frequency resolution up to a minimally achievable width set by the decoherence of the quantum computer. The circuit depth is measured by the number of (arbitrarily connected) two-qubit gates. (**C**) Optimal resolution for all three molecules. The circles indicate the resolution at optimal circuit depth, and the dashed black horizontal lines indicate the resolution accessible in NMR experiments.

The compounds are depicted in Fig. 3A and are taken from the example set of Spinach (*23*), an advanced classical simulation package that leverages decoherence in the NMR experiment to make the compuation more efficient (*4*). Each system can be simulated on a classical computer in several hours, provided access to 32 CPU cores, 128-GB random-access memory, and a graphics card as powerful as the Titan V. The interaction graphs characterizing the molecules’ nuclear spin Hamiltonians have a compact structure and are composed of strongly interacting clusters of four to seven spins that are weakly connected to other clusters. The compact nature of the interaction graphs—which give rise to rapidly spreading strong correlations—makes these systems hard to simulate on a classical computer, although these NMR experiments can be described without the long-range dipolar interactions that are central to other challenging NMR experiments such as solid-state NMR.

We estimate the resources required to simulate these systems using a quantum computer by using product formulas to prescribe circuits that implement time evolution under the Hamiltonian of Eq. 2. While there are many quantum algorithms that implement quantum dynamics, product formulas are considered to have the lowest resource overhead and be most suitable for early quantum devices (*7*, *24*). We exploit both the cluster structure of the nuclear interactions and inherent dephasing in the NMR experiment to further reduce the cost (see the Supplementary Materials). With the total number of qubits fixed to the number of nuclear spins, the performance crucially depends on the accuracy of the Hamiltonian engineering (Trotter error) and the fidelity with which each operation can be performed.

In Fig. 3B, we plot the achievable linewidth Δ*f* of the NMR spectrum as a function of the circuit depth *D* for quantum computers with various levels of noise, here, we model the decoherence and dephasing with a single parameter *F* that encapsulates the average fidelity of a gate. We assume that the time evolution quantum circuits are designed using a clustered first-order product formula (see the Supplementary Materials). We define the circuit depth as the number of (arbitrarily connected) two-qubit gates, as available in trapped-ion quantum computers (*9*). We observe a $1/\sqrt{D}$ scaling, reminiscent of the standard quantum limit, up to a critical depth where the decoherence of the quantum computer takes over. The improvement in resolution is due to a decrease in the Trotter error with circuit depth. At any given value of the gate fidelity *F*, there is an optimal circuit depth ∼1/log(1/*F*) arising from a competition between algorithmic error and decoherence, resulting in linewidth $\text{Δ}f∼\sqrt{\log(1/F)}$. Figure 3C depicts the expected optimal linewidth for all the molecules considered in this work. While we clearly observe that the larger molecules from Fig. 3A are considerably harder to simulate than the four spin methyl group that was computed here, it should be noted that these curves are expected to saturate for Hamiltonians corresponding to clustered molecules. To simulate the phosphorus cluster (Fig. 3A, iii) to the same level as the physical NMR experiment, we expect to require circuits of *O*(10^5^) gates with a typical gate infidelity of *O*(10^−4^), an infidelity that is two orders of magnitude better than the present experiment. These infidelities have been achieved in small trapped-ion systems (*25*, *26*), and future scaling strategies hold great promise for reaching the above performance metrics (*22*).

## DISCUSSION

Zero-field NMR experiments on molecules of similar size to those in Fig. 3A may thus be a context where prospective near-term quantum devices can show an advantage over classical computers. A more practically compelling context, however, is solid-state NMR. The same quantum algorithm described by Eq. 1 can be used to efficiently simulate the dynamics of solid-state NMR experiments after adding a dipolar interaction term to the Hamiltonian in Eq. 2 (see the Supplementary Materials). Magic angle spinning protocols can be modeled by endowing this dipolar term with time-dependant coefficients. Simulating solid-state NMR experiments, however, necessitates performing a powder average over 10^3^ to 10^4^ orientations of the system, each corresponding to an independent FID that must be computed. On classical computers, the simulation of each fixed orientation FID can be challenging for large systems such as membrane proteins or battery materials, thus making the overhead of powder averaged calculations onerous even after exploiting parallelization. The resource cost for quantum simulation of the powder-averaged FID for these systems, however, is roughly the same as the cost for computing a single FID corresponding to a fixed orientation.

Specifically, the sample complexity of the two cases is approximately equivalent on a quantum device. The FID computed for a fixed system orientation (Eq. 1), can be viewed as an estimator for a random variable corresponding to the total magnetization of the system (*8*). The powder-averaged FID can be computed by sampling a different orientation every time a term in Eq. 3 is sampled. Letting $E_{t;\text{Ω}}[M]$ be the expectation value corresponding to the FID at time *t* for a system in orientation Ω, the powder-averaged FID is simply $E_{\text{Ω}}[E_{t;\text{Ω}}[M]]$, with the outer expectation corresponding to a classical average over a uniform distribution of orientations. The variance of the estimator for the powder-averaged FID is

$$\mathrm{Var}[M]=E_{\text{Ω}}[{\mathrm{Var}}_{t,\text{Ω}}[M]]+{\mathrm{Var}}_{\text{Ω}}[E_{t;\text{Ω}}[M]]$$ (4)

The first term is the average quantum shot noise associated with the simulation of a fixed orientation FID and scales as *N*^2^ (*27*). The second term captures the classical noise associated with sampling the uniform distribution of orientations comprising the powder average. Empirically, at most, 10^3^ to 10^4^ such samples are typically required. For large systems consisting of *N* = 10^2^ to 10^4^ spins where quantum devices may prove advantageous over classical computers, the quantum shot noise dominates the classical noise. Therefore, roughly *N*^2^/ε repetitions (shots) of the quantum simulation suffice to achieve a precision ε for both fixed orientation and powder-averaged computations.

The resources required to simulate solid-state NMR experiments on quantum hardware is thus primarily determined by that required to simulate the dynamics of a single orientation of the system. While the hundreds to thousands of simulation-relevant spins in a large solid-state NMR samples may preclude computation of its dynamics on near-term quantum computers without any error correction, more mature quantum hardware may be able to perform the task.

Our zero-field demonstration provides the first proof of principle that quantum computers can simulate NMR spectra within experimental resolution and the experimentally demonstrated algorithm may eventually facilitate analysis of solid-state NMR experiments performed on systems of scientific and technological relevance. While scaling quantum NMR simulations to classically intractable systems will be challenging, it should be noted that the resource projections in Fig. 3 are substantially less demanding than most other near-term quantum computing applications (*7*, *28*, *29*). The physical reason behind the reduced resource cost is that dephasing is inherent in the dynamics of nuclear spin systems, with a rate given by the finite linewidth of spectral peaks in NMR experiments. Quantum simulations can tolerate decoherence in the quantum device as long as it is less than the dephasing rate of the spin system (*8*). We note that the noise characteristics in a quantum simulation platform will not typically be the same as an NMR experiment, as evidenced by the spurious peak seen in Fig. 1. If the noise of the simulation platform is well understood, then it can either be mitigated (*30*) or made to mimic the noise of the NMR experiment (*31*, *32*). If the noise of the platform is unknown, then techniques such as twirling can be used to turn it into an effective depolarizing channel (*33*, *34*), which is the channel used for the estimates in Fig. 3. In either case, as long as the finite magnitude of noise is sufficiently small, the platform can be used to faithfully simulate NMR experiments.

The hope for an advantage from these digital quantum simulations of NMR lies in time evolution on quantum hardware scaling with a lower-order polynomial of system size than state-of-the-art classical simulation packages such as Spinach. These classical methods also exploit decoherence in the NMR experiment to reduce the state space that is simulated from one scaling exponentially with system size to one scaling polynomially (*4*). The subsequent computation, extraction of the NMR spectrum from density matrix time evolution performed to machine precision using Taylor series expansions, is formally classically tractable but can prove practically challenging when high correlation orders must be kept in zero-field and solid-state NMR, corresponding to a state space scaling as a large polynomial of system size. Density matrix renormalization group (or tensor train) methods, which also exactly simulate density matrix time evolution on classical computers, have alternatively been applied in the context of NMR (*35*) but have struggled to simulate large systems due to the irregular three-dimensional interaction graphs that manifest between spins. Recent methodological developments, such as those discussed in (*27*), may be able to alleviate these issues to an extent, but it is yet unclear whether all simulation contexts will become tractable. Quantum Monte Carlo methods are another possible classical computing approach to simulating time evolution of quantum spin systems. These methods sample dynamical trajectories rather than exactly simulating density matrix dynamics and can, in principle, take advantage of decoherence in the NMR experiment and render the powder averaging overhead for solid-state NMR redundant similar to quantum hardware approaches. However, these methods remain largely unexplored in the context of NMR simulation. NMR thus provides a natural task where we can seek a practical quantum advantage from near-term quantum devices: simulation of noisy spin systems using noisy quantum computers.

## MATERIALS AND METHODS

NMR simulation circuits are run on a trapped ion quantum computer that uses the ^2^S_1/2_ states of ^171^Yb^+^ ions as the qubit states. We trap 15 ions in a chain for the simulation, and the circuits use four of those ionic qubits. Before each circuit iteration, ions are cooled using Doppler cooling and Raman sideband cooling and then reset to the logical 0 state via optical pumping. The qubit state is manipulated using 355-nm pulsed Raman beams. Single-qubit gates are implemented using SK1 pulses (*36*), and two-qubit gates are mediated by Mølmer-Sørensen interactions (*37*)—these gates are run sequentially. We measure the qubit states by shining 369-nm light resonant on the ^2^S_1/2_ → ^2^P_1/2_ cycling transition that scatters photons.

The time series data used to construct the NMR spectrum of acetonitrile were computed from 1000 shots of 102 different circuits, for each of which eight different initial basis states were prepared. Each circuit of 1000 shots took approximately 60 s to run. The total runtime to collect the data was therefore approximately 13.5 hours. Most of the runtime was taken up by classical compilation overhead. Improving the gate compilation procedure would lead to a reduction of runtime in the near term, thereby allowing faster computation of spectra.

While running circuits on the quantum machine, we perform system calibrations of trap voltages and gate amplitudes every hour to mitigate effects of system drift on circuit performance. We do not correct for state preparation and measurement (SPAM) errors in this study, and a table of our system’s SPAM characterization is presented in (*9*).

## Supplementary Materials

### This PDF file includes:

Supplementary Text Figs. S1 to S9 References
