# Supplementary material for: Digital quantum simulation of NMR experiments
Source: Sci Adv. 2023 Nov 17;9(46):eadh2594. doi: 10.1126/sciadv.adh2594 (PMC10656062; doi:10.1126/sciadv.adh2594)
Supplement: Supplementary file 1 — Supplementary Text Figs. S1 to S9 References [file sciadv.adh2594_sm.pdf]

Science Advances
AAAS

Supplementary Materials for

Digital quantum simulation of NMR experiments

Kushal Seetharam *et al.*

Corresponding author: Kushal Seetharam, kushalseetharam@gmail.com

Sci. Adv. **9**, eadh2594 (2023)
DOI: 10.1126/sciadv.adh2594

This PDF file includes:

Supplementary Text
Figs. S1 to S9
References

S1. SCALING METHODS TO LARGER SYSTEMS

Here, we give details of the circuit synthesis and compressed sensing techniques used in the experimentally demonstrated quantum simulation, and then discuss how they may be applied to simulations of larger systems.

A. Time-evolution circuit synthesis

We use the numerical optimization algorithm in Ref. [12] to synthesize the circuits implementing the time-evolution unitary $U(t) = \exp(-iHt/\hbar)$, with the Hamiltonian given in Eq. (S.1). The algorithm implements a bottom-up approach, building the single- and two-qubit gate decomposition of a n-qubit unitary by iteratively searching for a m-qubit gate decomposition with $m < n$. Initially, m is set to $n-1$. The algorithm is hardware topology and gateset aware; to specialize for the trapped-ion system, we allow all-to-all connectivity of qubit interactions and choose Mølmer-Sørensen gates with variable angles as the interaction gate.

We choose a unitary error of $\epsilon = 10^{-2}$, with the synthesis algorithm producing a circuit in terms of Mølmer-Sørensen (MS) gates and generic single-qubit rotations that approximates the true time-evolution unitary within this error. We then iteratively perform a X-Z-X decomposition of each single-qubit rotations, commuting the trailing X rotation through each MS gate before decomposing the next single-qubit rotation. This optimization results in roughly two Z rotations and two X rotations after each MS gate. As Z rotations are implemented virtually in the trapped-ion system, the final circuit has only two physical single-qubit rotations for each MS gate, and thus the physical circuit depth is reduced compared to the initial output of the synthesis algorithm. An example of the final optimized circuit is shown in Fig. S1. Typically, the produced circuits were composed of up to 40 MS gates and 80 physical single-qubit gates.

B. Compressed sensing

A general function in the frequency-domain that is nonzero in a specified frequency window can be reconstructed by Fourier transforming a corresponding time-domain signal that is uniformly sampled at the Nyquist rate. If the function is known to be sparse in the frequency domain, however, the time signal can be undersampled by choosing a non-uniform subset of time points which still capture the relevant information in the frequency domain [16]. The missing points on the original uniform time grid create artifacts in Fourier transform of the signal, however, which must then be removed using a compressed sensing reconstruction algorithm that exploits the assumed sparsity of the frequency signal. NMR spectra are often sparse as they are composed of a series of Lorentzian peaks, and therefore compressed sensing techniques allow for a dramatic reduction in the sampling required during an NMR experiment [11].

This sparsity can also be exploited in quantum simulations of NMR experiments by computing the FID at only the undersampled time points and then reconstructing the spectrum. We compute the FID at 102 out of the $N_s = 4096$ time points on the uniform grid, choosing the points according to a sine-weighted Poisson gap schedule. Such schedules have been shown to reduce undersampling artifacts [18]. The points are randomly chosen with the likelihood to pick a point $m+1$ on the uniform grid, given we have picked a point m, set by a Poission distribution with mean proportional to $\sin(\alpha\pi m/N_s)$. Specifically, we choose $\alpha = 0.5$, resulting in a schedule that is dense at short times before becoming increasingly sparse at later times. We find that this choice allows for much larger compression compared to a schedule with uniformly distributed gaps between points, or a schedule that is also dense at late times (corresponding to $\alpha = 1$). After computing the undersampled FID, we reconstruct the spectrum using the iterative soft thresholding (IST-S) algorithm [11].

In Fig. S2(a), we plot the FID computed via noisy emulations for all 4096 time points, and compare with the 102 points that were experimentally computed. This plot corresponds to Fig. 2(a) of the Main Text, but with the quantities depicted over the full time grid. In Fig. S2(b), we plot the spectrum computed after padding the experimental data with zeros for all time points that were not computed. We see that there is some signal at the spectral peaks expected from the noisy emulation, but the signal-to-noise is very large. The zero-padded spectrum in this plot corresponds to the green dots in Fig. 2(b) of the Main Text. In Fig. S2(c), we plot the compressed sensing reconstruction of the experimentally computed spectrum, and see that the signal-to-noise is dramatically improved. The reconstructed spectrum in this plot corresponds to the yellow curve in Fig. 2(c) of the Main Text.

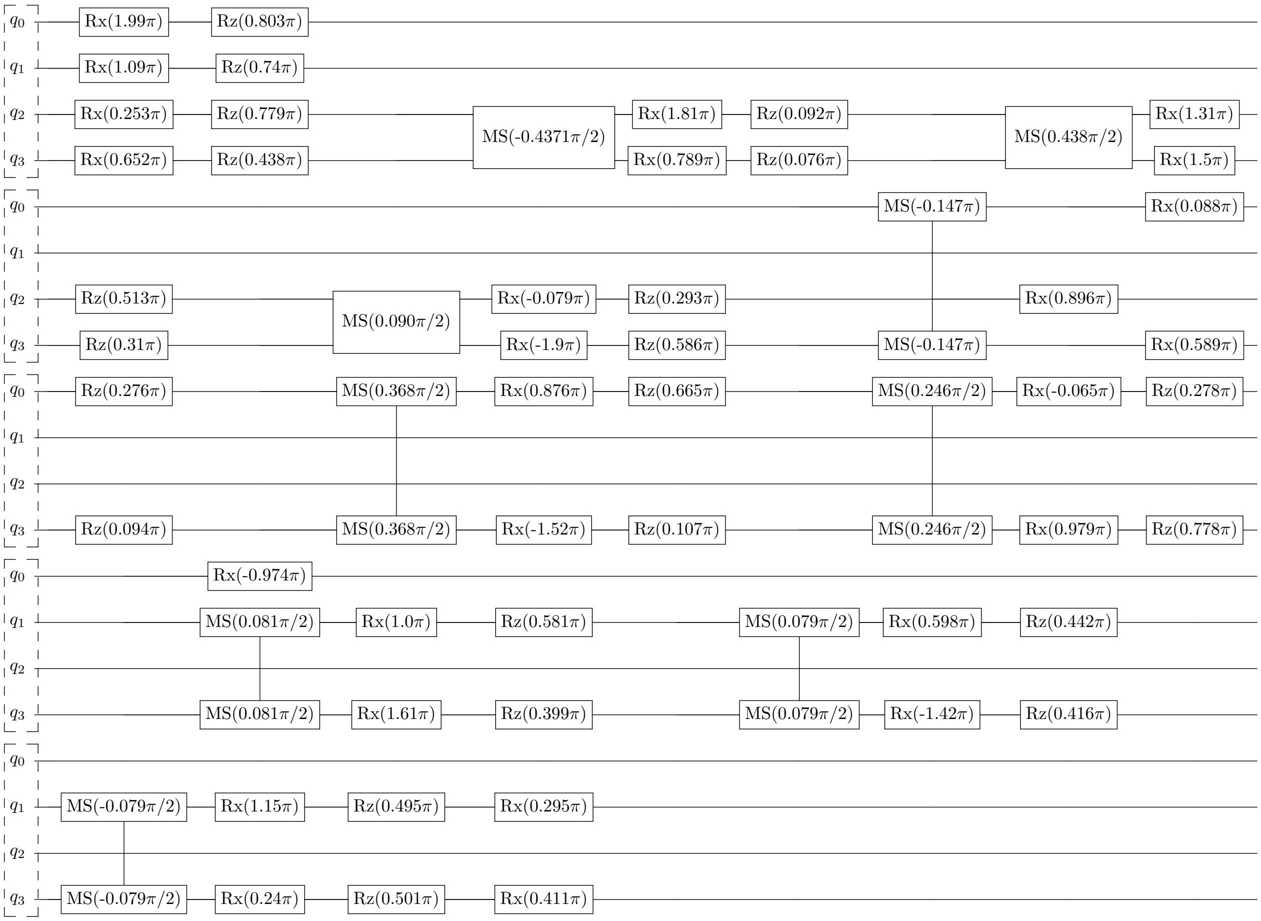

Figure S1: **Time-evolution circuit.** Example time-evolution circuit generated by numerical synthesis algorithm corresponding to $t = 0.07$ s. Circuit is split into five rows and read top to bottom, with the start of each row indicated by a dashed box around the four qubits in the experiment.

C. Scaling to larger systems

The numerical optimization algorithm we use is likely to be limited to producing time-evolution circuits for systems of up to ~ 7 spins [12]. This tool can still prove useful, however, when scaling to large, classically-intractable NMR simulations by exploiting the cluster structure of these molecules (see Fig. 3A of the Main Text). The strongly-interacting clusters are usually formed from 4-7 spins, and the optimization algorithm can be used to synthesize the time-evolution circuit for each cluster. These circuits can then be combined with a Trotter formula to implement the time-evolution of the entire systems [24]. Compared to a Trotter decomposition of the entire system, such a hybrid approach can reduce the overall circuit depth, as discussed in Sec. S3 B. Furthermore, at the level of discretization estimated in Fig. 3 of the Main Text, the simulation times are small enough that the optimization should converge very quickly, potentially enabling real-time compilation of the overall time-evolution circuit.

We note that numerical circuit synthesis of small subsystems and compressed sensing techniques form a synergistic combination of tools. For example, cluster-exploiting Trotter formulas allow for an overall reduction in resource cost at all simulation times, while compressed sensing non-uniform sampling schedules may sample more densely from short times where the resource cost is smallest. On the hardware side, the all-to-all connectivity of trapped ions makes them well-suited to the interaction graphs within clusters, and may allow comparatively smaller gate counts for the cluster evolution circuits. The relatively slow cycle time of ion devices is ameliorated by compressed sensing

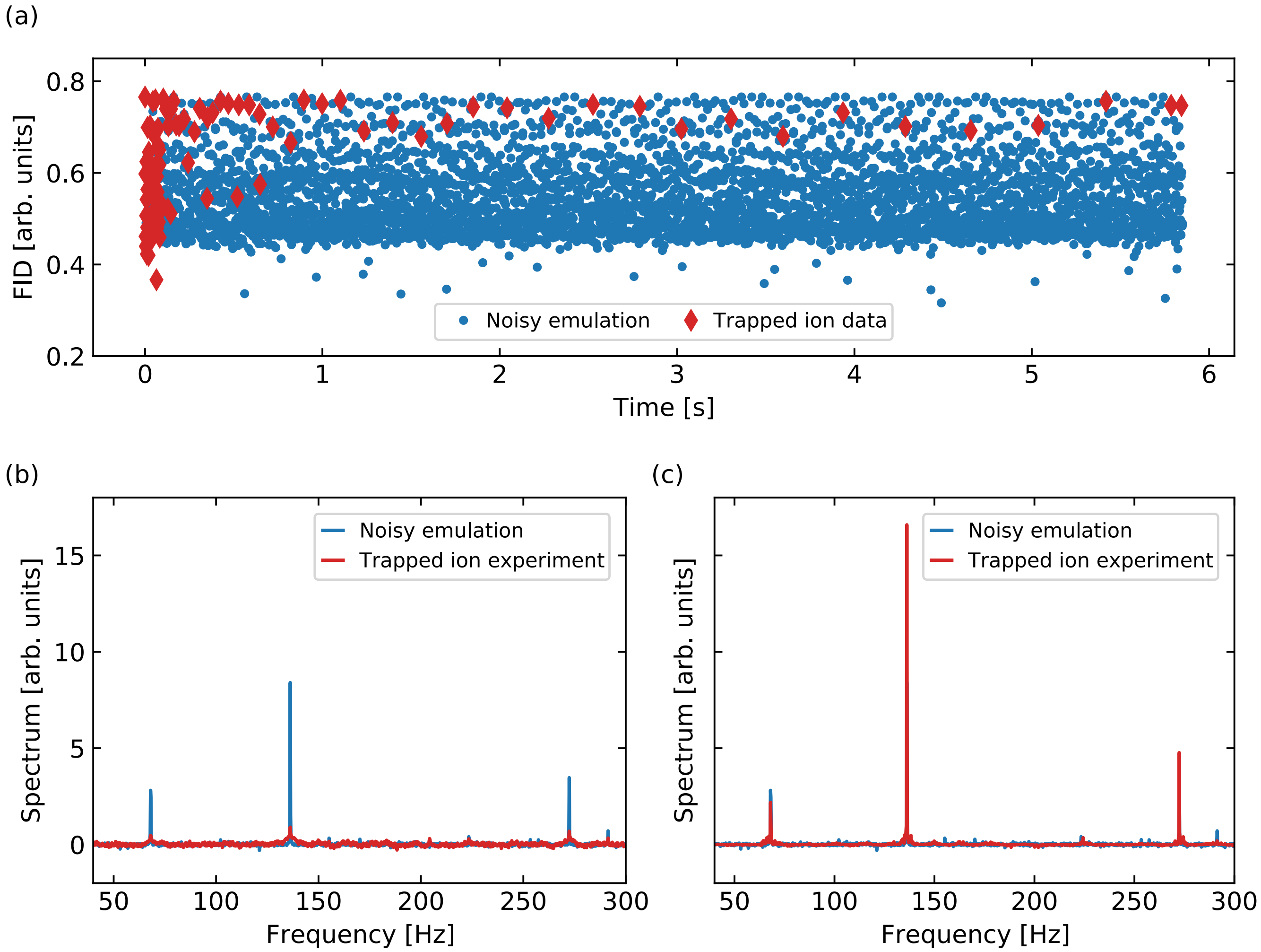

Figure S2: **Compressed sensing reconstruction.** **(a)** Comparison of the FID for noisy quantum circuit emulation on a fully sampled uniform time grid of 4096 points (blue circles) and the 99 data points experimentally measured on the ion trap device (red diamonds). **(b)** Fourier transform of the FID after replacing unsampled points with zeros. **(c)** Reconstructed spectrum after applying the iterative soft thresholding algorithm. The noise is modeled by two-qubit gates subject to both amplitude and phase damping with rates 0.005 and 0.035 respectively.

techniques, which reduce the number of time points that must be sampled. The combination of numerical circuit synthesis, which exploits the clustered interaction structure of a system, and compressed sensing, which exploits sparsity of the observable of interest in the transform domain, may similarly prove useful for quantum simulations in quantum chemistry and condensed matter systems where both of these characteristics are often present [14].

S2. SPECTRAL PEAK AT J/2

The acetonitrile spectrum we compute on the trapped ion quantum computer, depicted in Fig. 1 of the main text, exhibits a resonance at frequency $J/2$ which does not appear in the NMR experiment of Ref. [10]. Here, we explain the origin of this additional peak and discuss how to prevent such artifacts from appearing in future experiments.

The zero-field nuclear spin Hamiltonian of acetonitrile is

$$\hat{H} = J\left(\hat{\mathbf{S}}_1 + \hat{\mathbf{S}}_2 + \hat{\mathbf{S}}_3\right) \cdot \hat{\mathbf{S}}_4, \tag{S.1}$$

where $\{\hat{\mathbf{S}}_1, \hat{\mathbf{S}}_2, \hat{\mathbf{S}}_3\}$ represent the three ^{1}H and $\hat{\mathbf{S}}_4$ represents the ^{13}C. The eight positive magnetization states used to

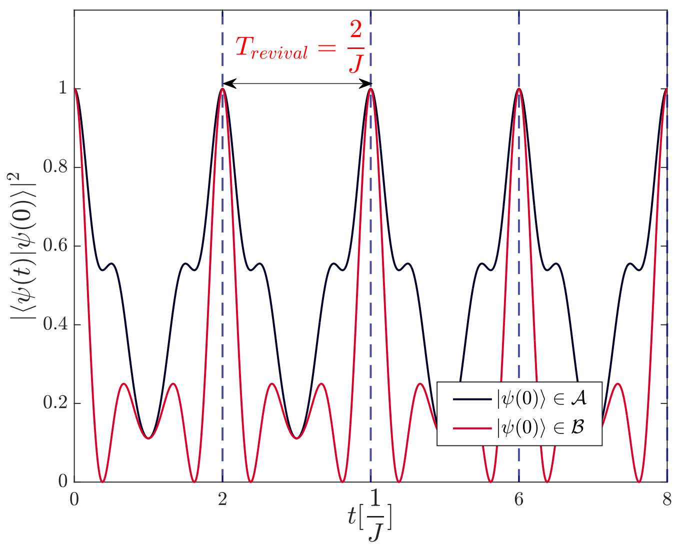

Figure S3: **Magnetization basis state revivals.** A system initialized in state $|\psi(0)\rangle$ selected from magnetization basis states $\mathcal{A} = \{\tilde{m}_1, \tilde{m}_3, \tilde{m}_5\}$ and $\mathcal{B} = \{\tilde{m}_4, \tilde{m}_6, \tilde{m}_8\}$ undergoes revivals with a period $2/J$.

compute the FID, see Eq. (3) of the main text, and their magnetizations are

$$
\begin{aligned}
|\tilde{m}_1 = 1.626\rangle &= |0000\rangle & \tilde{m}_1 &= 1.626\\
|\tilde{m}_2 = 1.374\rangle &= |0001\rangle & \tilde{m}_2 &= 1.374\\
|\tilde{m}_3 = 0.626\rangle &= |0010\rangle & \tilde{m}_3 &= 0.626\\
|\tilde{m}_4 = 0.374\rangle &= |0011\rangle & \tilde{m}_4 &= 0.374\\
|\tilde{m}_5 = 0.626\rangle &= |0100\rangle & \tilde{m}_5 &= 0.626\\
|\tilde{m}_6 = 0.374\rangle &= |0101\rangle & \tilde{m}_6 &= 0.374\\
|\tilde{m}_7 = 0.626\rangle &= |1000\rangle & \tilde{m}_7 &= 0.626\\
|\tilde{m}_8 = 0.374\rangle &= |1001\rangle & \tilde{m}_8 &= 0.374
\end{aligned}
$$

The small, four spin Hilbert space of the NMR active nuclear spins of the molecule along with the molecules highly symmetric nature—as codified by the single interaction scale J in Eq. (S.1)—combine to yield perfect revivals when the system is prepared in six of the above magnetization basis states. These states can be grouped into the triads $\mathcal{A} = \{\tilde{m}_1, \tilde{m}_3, \tilde{m}_5\}$ and $\mathcal{B} = \{\tilde{m}_4, \tilde{m}_6, \tilde{m}_8\}$ and we depict their revivals in Fig. S3. When viewed in the energy eigenstate basis, each of these six magnetization basis states only has weight on energy eigenstates whose eigenvalues are integer multiples of $J/2$. Consequently, all energies relevant to the dynamics are commensurate with a smallest splitting of $J/2$, which leads to the state reviving with perfect fidelity at this frequency. The revival of each state is mirrored in its entanglement dynamics. Each magnetization state begins in an unentangled product state, non-monotonically accrues entanglement over a period $T = \frac{2}{J}$, and then dis-entangles as it returns to the original product state. The high symmetry and small size of the molecule therefore causes the dynamics to defy usual expectations of ergodicity, with the entanglement of a system initially prepared in one of the states in $\mathcal{A}$ or $\mathcal{B}$ oscillating at a frequency $J/2$ instead of growing monotonically in time.

The numerical optimization algorithm, Ref. [12], we use to synthesize time-evolution circuits for each time point reflects this oscillating entanglement in the gate depth of the synthesized circuits. Specifically, times at which the system is more heavily entangled correspond to deeper circuits with a larger number of two-qubit gates, as can be seen in Fig. S4.

Noise in the system affects deeper circuits more than shallower ones, and therefore imprints the $J/2$ entanglement oscillation onto the experimentally measured observable by lowering the fidelity of the signal at this frequency. We can gain visibility into this process by computing the average Bhattacharyya coefficient (BC) between the measured basis state populations and noiseless emulations of the circuits for each time point. The BC is defined as

$$\mathrm{BC} = 1 - \frac{1}{2}\sum_j \left(\sqrt{p(j)} - \sqrt{q(j)}\right)^2, \tag{S.2}$$

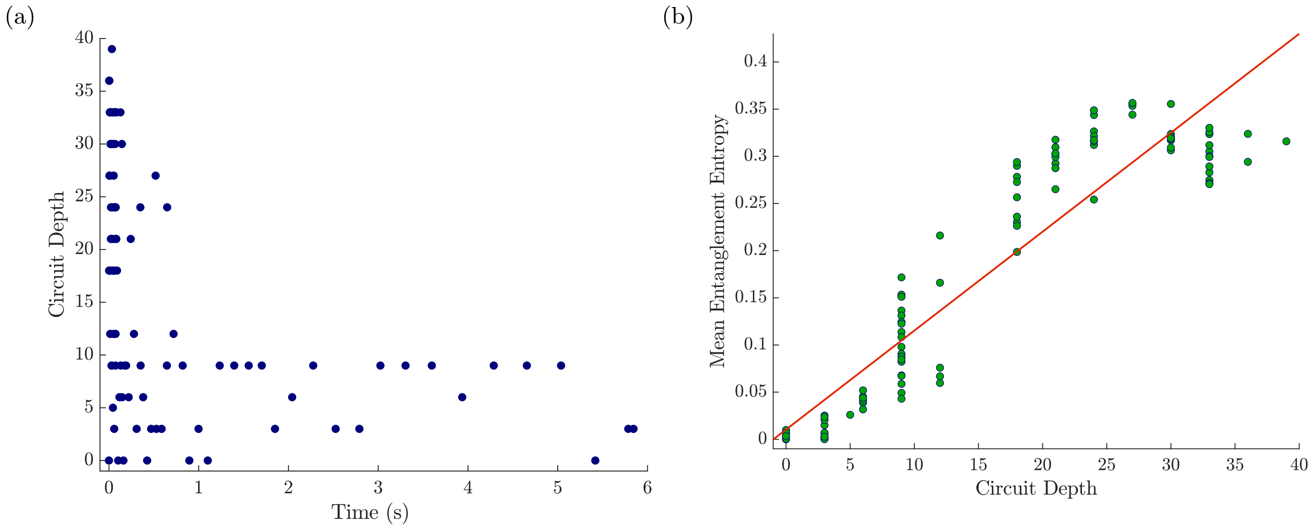

Figure S4: **Synthesized circuit depth and entanglement.** **(a)** Two-qubit gate count of synthesized time-evolution circuits for each FID evolution time measured on the trapped-ion device. **(b)** Entanglement entropy of the system at a particular evolution time compared to the two-qubit gate count of the circuit implementing that evolution. We average the final entanglement entropy for systems initialized in each of the eight magnetization basis states used to compute the FID.

where j runs over all computational basis states, and $p(j)$ and $q(j)$ are the corresponding occupation probabilities, given by the diagonal elements of the density matrix, of the two states beings compared. The BC gives a measure of the fidelity of the experimental runs and we plot it for every experimentally measured time point in Fig. S5(a). We see that it varies as a function of time, and these oscillations correspond to time-evolution circuits that have a larger two-qubit gate count as shown in Fig. 2C of the Main Text. In Fig. S5(b), we use the same compressed sensing algorithm used to compute the NMR spectrum to reconstruct the Fourier transform of the the BC dynamics depicted in Fig. S5(a). We observe a sharp peak at $J/2$, confirming that the fidelity of the experimental oscillates at a frequency of $J/2$. These oscillations corresponding to oscillating depths of the synthesized circuits, which in turn reflect the entanglement revivals of the molecule's underlying dynamics. The above story confirms why noisy circuit simulations, such as the one depicted in Fig. 2A of the main text, also exhibit this $J/2$ resonance peak in the computed NMR spectrum regardless of the type of decoherence channel used to simulate noise.

artifact peaks such as the $J/2$ resonance can easily be removed in future experiments. By padding all time-evolution circuits so that they have roughly the same depth as the deepest synthesized circuit, the noise in the system can no longer imprint any frequency on the measured signal as the gate depths no longer oscillate. We show in Fig. S6 that such padding dramatically decreases the height of the $J/2$ peak in noisy circuit simulations of the experiment, where noise is modeled as a depolarizing channel on the gates. The padding will, however, slightly decrease the overall fidelity of the computed FID as every point will be subject to as much noise as the deepest time-evolution circuit. If we desire to compute the maximum fidelity signal allowable by hardware, we can compute FID twice - once with padded circuits and once without. Any feature that vanishes in the padded experiment can be removed from the higher fidelity non-padded experiment.

Lastly, we note that artifact peaks are unlikely to appear during quantum simulations of the majority of NMR experiments, and become increasingly unlikely when scaling to classically intractable systems. Small molecules which do not exhibit the high degree of symmetry exhibited in Eq. (S.1) are unlikely to exhibit the dynamical revivals at the heart of artifact peaks. Larger systems, including those with some symmetry, are even less likely to exhibit revivals as entanglement spreads throughout the system. In fact, classically intractable systems are intractable precisely because quantum correlations spread quickly throughout the system. Furthermore, quantum simulation algorithms that generalize to larger systems, such as product formulas, typically have gate depths that monotonically increase with the simulation time. This relationship is also directly true for analog quantum simulation. Noise in these cases will lead only to a broadening of spectral peaks, and cannot imprint any artifact frequencies on the measured signal. Therefore, artifact peaks are unlikely to be a common concern during quantum simulations of NMR experiments. Even in small, highly symmetric systems where the peaks might appear, circuit padding is a cheap way to remove these artifacts.

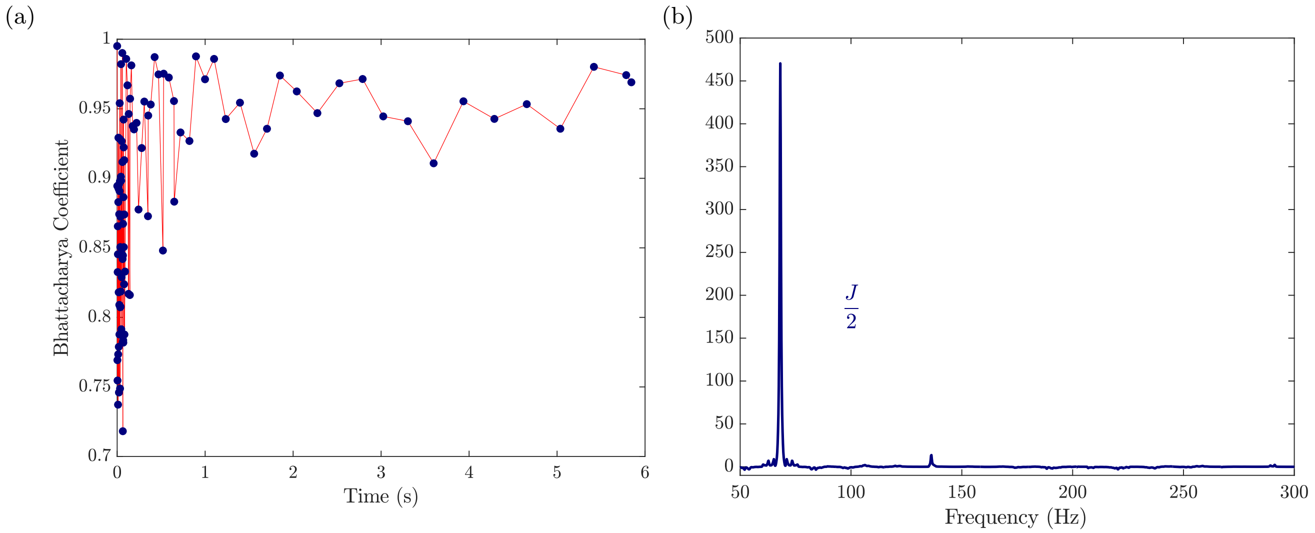

Figure S5: **Bhattacharyya coefficient between trapped ion measurements and noiseless emulation of the experiment.** **(a)** BC vs evolution time. **(b)** Compressed sensing reconstruction of the frequency spectrum of the BC. We see that the BC, a measure of the fidelity of the system, only varies at the frequency $J/2$ with which the system's entanglement, and therefore circuit depth, oscillates.

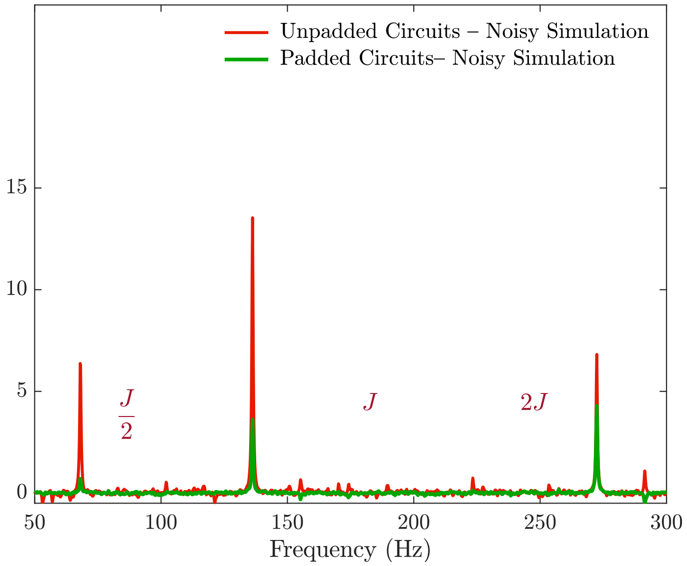

Figure S6: **Noisy circuit simulation.** The zero-field NMR spectrum of acetonitrile computed using noisy circuit simulations with and without padding. The padded circuits no longer have depths that oscillate in accordance with the system's entanglement, and therefore do not exhibit the artifact peak at $J/2$. Noise was simulated by by adding a depolarizing channel to each gate, with a rate of 10^{-3} for single-qubit gates and 10^{-2} for two-qubit gates.

S3. RESOURCE ESTIMATES FOR NMR SIMULATION

The standard Hamiltonian simulation task (e.g. by Suzuki-Trotter product formula, hereafter product formula), seeks to approximate exact unitary dynamics within a finite precision ϵ (clarified below). However, the spectral resolution—alternatively, the line-width—of NMR experiments are set by the dephasing of the nuclear spins. The inherent dephasing in the experiments we seek to simulate reduces the resource cost simulation compared to purely coherent systems. In what follows, we show how this discrepancy between the standard Hamiltonian simulation task (simulating a unitary to finite *precision*) and the task of employing Hamiltonian simulation as a sub-routine to compute an NMR spectrum (with finite *spectral resolution*) can yield gate counts that are several orders of magnitude

smaller for the latter task, making it tractable on NISQ devices. To perform Hamiltonian simulation, we proceed by using a variant of first-order product formula that exploits the clustered structural motif present in many molecules that are classical challenging to simulate. In what follows, we first elucidate the distinction between the standard Hamiltonian simulation task and the task at hand and provide bounds on the requisite two-qubit gate fidelity and gate counts for computing NMR spectra with finite resolution. We round out our discussion by providing commutator bounds relevant to estimating the quantum resources required to simulate the NMR spectra of molecules with a clustered interaction structure.

A. Approximating Hamiltonian Dynamics for NMR Simulations

We begin by clarifying the difference between a standard Hamiltonian simulation task and the task of using Hamiltonian simulation to simulate NMR experiments. We consider performing Hamiltonian simulation via first order Trotterization, a simple but powerful and gate efficient method for simulating Hamiltonian dynamics. Keeping our discussion somewhat general for the moment, let's say we have a Hamiltonian $H = \sum_\mu h_\mu$, composed out of a number of N_c terms h_μ. We'd like to replace the time-evolution operator $U = e^{-i\Delta t H}$, for some small time-step Δt by our simple product formula:

$$\tilde{U} = \prod_{\mu=1}^{N_c} e^{-i\Delta t h_\mu}.$$

It follows from Baker-Campbell-Hausdorff (BCH), by keeping only lowest order contributions in Δt, that

$$\|U - \tilde{U}\| \leq \frac{(\Delta t)^2}{2} \sum_{\mu=1}^{N_c} \| \sum_{\nu>\mu} [h_\nu, h_\mu]\|, \tag{S.3}$$

as obtained in Ref. [7]. Consequently, we could also write the fidelity of the simulation as

$$\mathcal{F}_{\delta t} = \|U\tilde{U}\| \geq e^{-\beta(\Delta t)^2/2}, \quad \text{where} \quad \beta = \sum_{\mu=1}^{N_c} \| \sum_{\nu>\mu} [h_\nu, h_\mu]\| \tag{S.4}$$

Let's say we'd like to evolve for a total time $T = r\Delta t$, then the total fidelity will be

$$\mathcal{F}_{PF}(T) = \prod_{i=1}^{r} \mathcal{F}_{\delta t_i} = e^{-r\beta(\delta t)^2/2} = \exp\left(-\frac{\beta T^2}{2r}\right), \tag{S.5}$$

If we want a precision ϵ, then we need $\mathcal{F} = 1 - \epsilon \approx e^{-\epsilon}$. This sets the Trotter number r to achieve a precision ϵ:

$$r_\epsilon = \frac{\beta T^2}{2\epsilon}. \tag{S.6}$$

Achieving this can be quite challenging for even intermediate times, in particular in NISQ settings.

The task of simulating the relevant dynamics corresponding to an NMR experiment does not, however, require the approximation of unitary dynamics generated by the Hamiltonian to finite, time-independent precision ϵ. It requires instead the simulation of a spectrum to finite spectral resolution Δf. In an NMR experiment, $\Delta f \sim \gamma$, the dephasing rate of a single nuclear spin in experimentally interrogated sample. Thus, the task of simulating an NMR experiment with resolution Δf is equivalent to simulating Hamiltonian dynamics of a sample of N spins in which each spin decoherences independently with an *effective* dephasing rate $\gamma = \frac{\Delta f}{2\pi}$. Such dephasing exponentially degrades the fidelity, vis-a-vis perfect unitary dynamics given by the Hamiltonian, as $\mathcal{F}_{NMR} \sim e^{-\gamma N t}$. Thus, there is a subtle but essential distinction between the task of approximating a unitary to multiplicative error ϵ and performing Hamiltonian simulation to compute a spectrum with finite spectral resolution Δf. If, for the moment, we neglect decoherence in our quantum hardware and consider only algorthmic error due to our product formula, as given in S.5, then setting $\mathcal{F}_{PF}(T) = \mathcal{F}_{NMR}(T)$ we find a upper bound for the minimal necessary Trotter number:

$$r_{NMR} = \frac{\beta T}{2\gamma N}. \tag{S.7}$$

As typical experiments interrogate regimes up to where $\gamma T \sim 1$, the number of Trotter steps r is reduced by a factor of $\frac{N}{\epsilon}$ as compared to the case of fixed precision—for the examples examined in the Main Text, this corresponds to a $O(10^3 - 10^4)$-fold decrease in the number of steps required to realize the longest dynamics relevant to experiment.

For a more careful estimate of the necessary resources, we also account for the decrease in fidelity due to decoherence in our quantum hardware. We describe the decaying fidelity of our experiment due to hardware error as $\mathcal{F}_h(T) \sim F^{rN_g}$, where F is the two-qubit gate fidelity, r is the Trotter number, and N_g is the number of two-qubit gates required to realize a particular Trotter step. Note that by such a description, we assume that hardware error and algorithmic error are independent of each other and that two-qubit gates dominate the hardware error. The product of $\mathcal{F}_h(T)$ and $\mathcal{F}_{PF}T$ must be greater than or equal to $\mathcal{F}_{NMR}$ in order to perform reliable simulation. We obtain, the following requirement:

$$-N_g \log(F) + \beta(\Delta t)^2/2 \leq \gamma N \Delta t. \tag{S.8}$$

This will have a solution for the Trotter time step Δt as long as

$$F \geq e^{-\gamma N \Delta t / N_g}. \tag{S.9}$$

Requiring our quantum simulation fidelity to match the fidelity of the experiment —thereby recasting S.8 as an equality —and re-arranging, we can establish an equation for $\gamma \sim \Delta f$:

$$\gamma = \frac{1}{N}\left(\frac{\Delta t \beta}{2} - \frac{N_g \log(F)}{\Delta t}\right) \tag{S.10}$$

By optimizing over the Trotter step Δt, we can set the ultimate resolution Δf_{opt} of our experiment:

$$\Delta f_{opt} = \frac{\gamma_{opt}}{2\pi} = \frac{1}{2\pi N}\sqrt{2N_g \beta \log(1/F)} \tag{S.11}$$

Note that this optimal resolution is a simple function of the fidelity of the quantum hardware employed, given by F, and the efficiency of the algorithm used, as encoded by β and N_g. On the hardware side, improving gates and thereby improving the gate fidelity, parametrized by F, would lower the resolution. Similarly, on the algorithmic side, finding more efficient circuits to realize a single Trotter step (lowering N_g) or better product formulae (lowering β), would improve the resolution—in what follows, we provide strategies on how to achieve both of these algorithmic improvements.

B. Commutator Bounds for Clustered Hamiltonians

As implied by Eq. (S.11), decreasing β (defined above in Eq. (S.4)) small would improve the resolution of the simulation. In what follows, we compute β, assuming an NMR Heisenberg Hamiltonian with clustered interactions, first in the standard way and then by taking advantage of the cluster motif. We show that by doing the latter, we can reduce β substantially.

Before beginning this program, it is useful to establish some intuition for how clustered interactions reduce β vis-a-vis the case of all-to-all couplings. If we take an all-to-all model with some typical coupling $J/\sqrt{N}$ (taking into account Kac normalization to keep the energy extensive in the system size N), we find

$$\beta = O(J^2N^2), \quad \text{such that} \quad r \sim \left(\frac{JN}{\gamma}\right)^2 (\gamma T). \tag{S.12}$$

The situation changes for clustered Hamiltonians where each spin typically interacts with a sub-extensive number of spins k. For each term in the latter, only k terms contribute in the commutator and there are kN terms, yielding:

$$\beta \sim k^2 N J^2, \quad \text{such that} \quad r \sim \left(\frac{Jk}{\gamma}\right)^2 (\gamma T). \tag{S.13}$$

Note that, due to the clustered nature of the interactions, r does not scale with N: Increasing the number of spins does not increase the Trotter number.

Having established an heuristic derivation for the scaling of β and thereby the Trotter number, we turn to the present situation of a Heisenberg model with local fields.

$$H = \sum_{ij} J_{ij}(S_i^x S_j^x + S_i^y S_j^y + S_i^z S_j^z) + \sum_i h_i S_i^z. \tag{S.14}$$

where all terms can be labeled by $\mu = (i, j, \sigma)$, indicating the bond and the operator that is acted with. If we perform a scheme which alternates all Ising-XX gates with all Ising-YYs and all Ising-ZZs, then β is comprised of three terms, defined below: β_1, β_2, β_3.

We first bound β_1, given by:

$$\beta_1 = \sum_{ij} |J_{ij}| \| \sum_{kl} J_{kl}[S_k^y S_l^y + S_k^x S_l^x, S_i^z S_j^z]\|, \tag{S.15}$$

Straightforward algebra brings us to

$$\beta_1 = \sum_{ij} |J_{ij}| \| \sum_k \left(2J_{ki}(S_k^x S_i^y - S_k^y S_i^x)S_j^z + 2J_{kj}(S_k^x S_j^y - S_k^y S_j^x)S_i^z \|\right). \tag{S.16}$$

where the fact that $J_{ij} = J_{ji}$ is used. We therefore have:

$$\beta_1 \le \sum_{ij} 2|J_{ij}| \sum_k \left(\|J_{ki}(S_k^x S_i^y - S_k^y S_i^x)S_j^z + J_{kj}(S_k^x S_j^y - S_k^y S_j^x)S_i^z \|\right), \tag{S.17}$$

which can again be bounded as:

$$\beta_1 \le 2\sum_{ij} |J_{ij}| \sum_k \left|J_{ki}\right| \|(S_k^x S_i^y - S_k^y S_i^x)S_j^z \| + |J_{kj}| \|(S_k^x S_j^y - S_k^y S_j^x)S_i^z \|\big). \tag{S.18}$$

Computing the norm directly—$\|(S_k^x S_i^y - S_k^y S_i^x)S_j^z\| = 1/4$ —we arrive at:

$$\beta_1 \le \sum_{ijk} |J_{ik}||J_{ki}| \tag{S.19}$$

We can similarly bound β_2, which is given by:

$$\beta_2 = \sum_{ij} |J_{ij}| \| \sum_{kl} J_{kl}[S_k^y S_l^y, S_i^x S_j^x]\|, \tag{S.20}$$

as

$$\beta_2 \le \frac{1}{2} \sum_{ijk} |J_{ik}||J_{kj}| \tag{S.21}$$

Finally there are local field terms, which if we do them together with the ZZ gates would give

$$\beta_3 = \sum_i |h_i| \| \sum_{kl} J_{kl}[S_k^y S_l^y + S_k^x S_l^x, S_i^z]\|, \tag{S.22}$$

which gives

$$\beta_3 = 2\sum_i |h_i| \| \sum_k J_{ki}(S_k^x S_i^y - S_k^y S_i^x)\|, \tag{S.23}$$

and thus

$$\beta_3 \le \sum_{ij} |h_i||J_{i,j}| \tag{S.24}$$

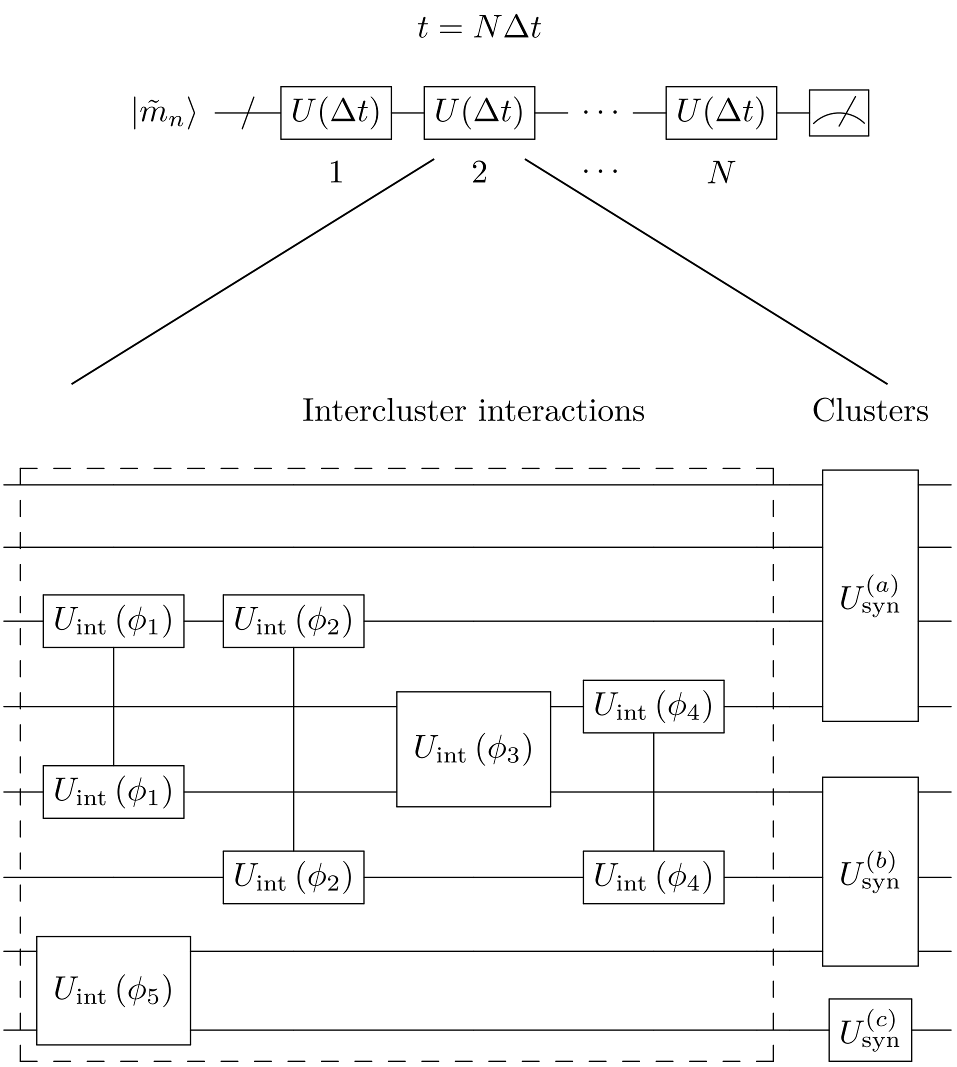

Figure S7: **Cluster Trotter circuit.** Schematic of quantum circuits simulating the time-evolution of challenging NMR systems. The total time-evolution is split into N identical increments. Each increment is composed of numerically synthesized circuits enacting the time-evolution of strongly interacting clusters of spins, along with two-qubit gates enacting interactions between spins from different clusters. An example of a numerically synthesized circuit is given in Fig. S1.

However, one can obtain a tighter bound by treating the Z-gates as a single global gate. Doing so yields:

$$\tilde{\beta}_3 = \| \sum_{kl} J_{kl} [S_k^y S_l^y + S_k^x S_l^x, \sum_i h_i S_i^z] \|, \tag{S.25}$$

in which case we'd arrive at

$$\tilde{\beta}_3 \leq \frac{1}{2} \sum_{ij} |h_i - h_j| |J_{i,j}| \leq \beta_3. \tag{S.26}$$

Therefore, we have that the total spectral norm of the nested commutator β is bounded by:

$$\beta \leq \beta_1 + \beta_2 + \tilde{\beta}_3 \leq \frac{3}{2} \sum_{ijk} |J_{ik}| |J_{kj}| + \frac{1}{2} \sum_{ij} |h_i - h_j| |J_{i,j}|. \tag{S.27}$$

For many molecules, the interactions between nuclear spins follows a clustered motif. In particular, we examine the computational resources to simulate classically hard molecules that are composed of strongly-interacting clusters

tethered together with weakly interacting links. We leverage the clustered motif by performing a variant of first-order Trotter formula wherein the dynamics of the small clusters are numerically synthesized to high precision, using for example the algorithm of Ref. [12], while weak interactions between such clusters are rendered via a pairwise Trotter decomposition of the Hamiltonian. A quantum circuit schematic depicting such a cluster-exploiting Trotter decomposition is given in Fig. S7.

To understand how this modifies β, we consider the following Hamiltonian:

$$H = H_c + \sum_{i,j} J_{i,j}(S_i^x S_j^{x,c} + S_i^y S_j^{y,c} + S_i^z S_j^{z,c}) + \sum_i h_i S_i^z, \tag{S.28}$$

and

$$H_c = \sum_{k,l} V_{k,l}(S_k^{x,c} S_k^{x,c} + S_k^{y,c} S_l^{y,c} + S_k^{z,c} S_l^{z,c}) + \sum_k h_k S_k^{z,c}, \tag{S.29}$$

where the superscript simply indicates that the operator belongs to the cluster H_c. If we could synthesize the Hamiltonian of the cluster efficiently (see below), we find:

$$\beta \leq \frac{3}{2}\sum_{i,j,k} |J_{i,k}V_{k,j}| + \frac{3}{2}\sum_{i,j,k} |J_{i,k}J_{k,j}| + \frac{1}{2}\sum_{i,k} |J_{i,k}||h_k - h_i|. \tag{S.30}$$

By comparing Eq. (S.30) with Eq. (S.27), we see that the latter avoids terms with intra-cluster couplings (i.e. terms like $V_{i,j}V_{k,l}$). For the cases considered in the Main Text, making use of the cluster structure in this manner reduces the Trotter number by one to two orders of magnitude.

We note that both the complexity of the coupling graph as well as number of coupling terms that determine the Trotter error, thus allowing for cases where a molecule with a simpler coupling graph may have less error than a slightly smaller molecule with a more complicated coupling graph. Indeed, this is seen in Fig. 3 of the Main Text.

S4. CORRELATION SPREADING FROM NMR HAMILTONIANS

While NMR was originally proposed as a platform for quantum computing, the association of quantum speedup with quantum entanglement has raised questions about the possibility of speedup in NMR based quantum computing. It has been shown that the state of the system is always separable [39], severely restricting the accessible phase space. While large entanglement in pure states precludes efficient classical simulation, large operator entanglement can do the same for mixed states if one has access to two-time correlation function. This is exactly the setting of standard NMR spectroscopy. As far as we know, the argument was first laid out in [40] and called strong correlations, for lack of a better word. For completeness, we repeat the arguments of Ref. [40] for our specific setup, i.e. we have computed the operator entanglement entropy and Schmidt rank for the S^z_{tot}-magnetization evolving under a few relevant Hamiltonians, including two real molecules and some random matrices, see Fig. S8.

In complete analogy with the entanglement entropy for a state, we can partition are system in two subsystems A and B, and define a complete set of orthonormal basis operators on each subsystem A_i and B_i respectively. Any operator can than be decomposed into

$$O = \sum_i \lambda_i A_i \otimes B_i. \tag{S.31}$$

We define the operator entanglement as

$$S = -\sum_i |\lambda_i|^2 \log_2(|\lambda_i|^2). \tag{S.32}$$

The initial magnetization

$$S^z_{\mathrm{tot}} = \sum_i S_i^z = \sum_{i\in A} S_i^z \otimes \mathbb{I}_B + \sum_{i\in B} S_i^z \otimes \mathbb{I}_A, \tag{S.33}$$

has Schmidt rank 2 with respect to any bi-partition of the system. As shown in Fig. S8 the operator entanglement, in realistic systems, grows fast when this operator is evolved unitarily in time. It saturates at a value which is close to the

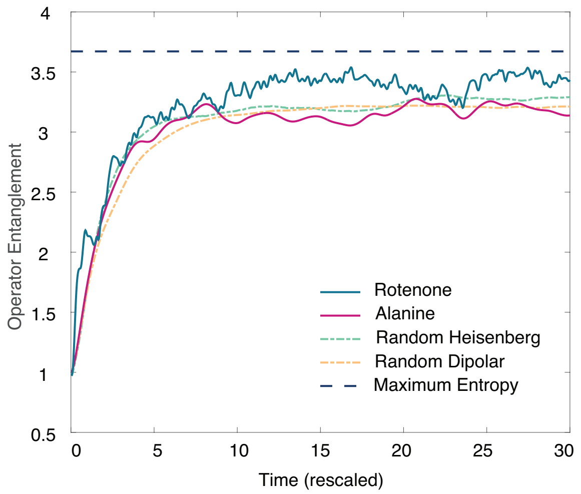

Figure S8: **Operator Entanglement I.** After a quench the operator entanglement in the total magnetization grows from its initial value $S = 1$ (Schmidt rank 2) to a value that is close to the maximal allowed entanglement. We show the evolution of two different molecules during an NMR spectroscopy sequence. For comparison we also show the operator entanglement in the corresponding random Hamiltonians, i.e. Hamiltonians where the couplings between the nuclear spins are drown from a Gaussian random matrix. The results are comparable, indicating fast spreading of correlations during NRM spectroscopy.

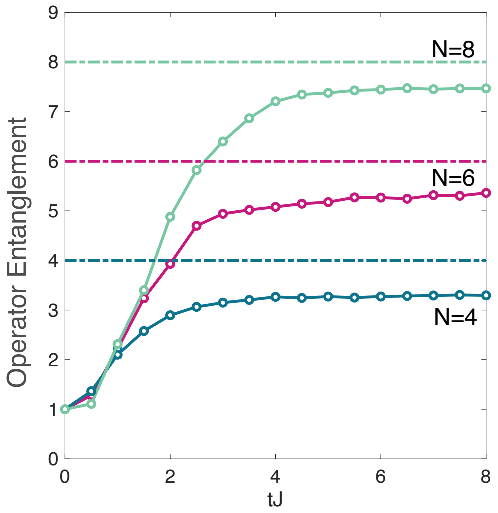

Figure S9: **Operator Entanglement II.** System size scaling of the operator entanglement in the time-evolved z-magnetization between a random bi-partition in a random Heisenberg model. The results have been averaged over 100 disorder realizations.

maximal allowed entropy. The large, and ultimately extensive (see Fig. S9), operator entanglement implies that the unitary dynamics becomes classically intractable. While this highlights the complexity of quantum dynamical systems at infinite temperature, it should be noted that real systems aren't isolated and the environment induced decoherence tends to destroy the operator entanglement structure. Whether or not the particular evolution is classicaly hard depends on the balance between the entanglement growth and the decoherence, e.g. recent development in tensor networks have lead to the possibility of simulating noisy quantum systems when the noise levels exceed a minimal threshold [41].

S5. SOLID-STATE NMR SIMULATION

The effective Hamiltonian for a specific orientation Ω of a system studied using solid-state NMR can be expressed as

$$H = H_0 + H_\Omega \tag{S.34}$$

where

$$H_0 = \omega_0 S^z_{\text{tot}} \tag{S.35}$$

with represents the global Zeeman term corresponding to the external magnetic field, assumed to be aligned with the z-axis, and

$$H_\Omega = \omega_0 \sum_i \omega_i \mathbf{e}^{(\Omega)}_{\text{cs}} \cdot \mathbf{S}_i + \sum_{i,j} b_{ij} \left(3(\mathbf{S}_i \cdot \mathbf{e}^{(\Omega)}_{\text{ij}})(\mathbf{S}_j \cdot \mathbf{e}^{(\Omega)}_{\text{ij}}) - \mathbf{S}_i \cdot \mathbf{S}_j \right) \tag{S.36}$$

represents the orientation-dependent chemical shifts induced by the external field and direct dipolar interactions between spins [1]. The Zeeman frequency, $\omega_0 = -\gamma_i B_{\text{ext}}$, depends on the gyromagnetic ratios, γ_i, of the spins and the strength, B, of the external magnetic field. For illustration purposes, we assume a homonuclear system where $\gamma \equiv \gamma_i$ is the same for all spins. We allow the external field pointing in the z-direction to define the lab frame and can express the orientation of the system as $\Omega = (\alpha, \beta, \gamma)$, where α, β, and γ are Euler angles that define the rotation of the system frame to the lab frame. The chemical shifts, with magnitudes $\omega_0\omega_i$ and directions $\mathbf{e}^{\Omega}_{\text{cs}}$, are most naturally derived starting from the principal axis frame of spin i. In this frame, the magnetic field induced in around the spin is parallel to the direction of the external field:

$$\mathbf{B}^{(P)}_i = \overline{\delta}^{(P)}_i \cdot \mathbf{B}^{(P)}_{\text{ext}} \tag{S.37}$$

where

$$\overline{\delta}^{(P)}_i = \begin{bmatrix} \delta^{XX}_i & 0 & 0 \\ 0 & \delta^{YY}_i & 0 \\ 0 & 0 & \delta^{ZZ}_i \end{bmatrix} \tag{S.38}$$

and $\mathbf{B}^{(P)}_{\text{ext}} = B_{\text{ext}} \mathbf{e}^{(P)}_{\text{ext}}$ is the external field expressed in the principal axis system. We can then apply the rotation matrices $\overline{R}(\Omega_i)$ and $\overline{R}(\Omega)$ to move from the principal axis frame to the system frame and from the system frame to the lab frame respectively. We can then write the induced field in the lab frame as

$$\mathbf{B}_i = B_{\text{ext}} \overline{\delta}_i \cdot \mathbf{e}_{\text{ext}} \tag{S.39}$$

where

$$\overline{\delta}^{(\Omega)}_i = \overline{R}^\dagger(\Omega) \cdot \overline{R}^\dagger(\Omega_i) \cdot \overline{\delta}^{(P)}_i \cdot \overline{R}(\Omega_i) \cdot \overline{R}(\Omega) \tag{S.40}$$

$$\mathbf{e}_{\text{ext}} = \overline{R}^\dagger(\Omega) \cdot \overline{R}^\dagger(\Omega_i) \cdot \mathbf{e}^{(P)}_{\text{ext}} \cdot \overline{R}(\Omega_i) \cdot \overline{R}(\Omega). \tag{S.41}$$

Note that Ω is determined by setting $\mathbf{e}_{\text{ext}} = \mathbf{e}_z$. Recognizing that $\overline{\delta}^{(\Omega)}_i \cdot \mathbf{e}_{\text{ext}}$ is a vector, we define its magnitude and direction as ω_i and $\mathbf{e}^{(\Omega)}_{\text{cs}}$ respectively via

$$\omega_i \mathbf{e}^{(\Omega)}_{\text{cs}} \equiv \overline{\delta}^{(\Omega)}_i \cdot \mathbf{e}_z. \tag{S.42}$$

The Hamiltonian term resulting from the induced field on spin i is

$$H^{\text{cs}}_i = -\gamma_i \mathbf{B}_i \cdot \mathbf{S}_i = \omega_0 \omega_i \mathbf{e}^{(\Omega)}_{\text{cs}} \cdot \mathbf{S}_i, \tag{S.43}$$

which we see depends on the system orientation Ω. The second term in Eq. (S.34) is a direct dipolar interaction between every pair of spins determined by the dipole vector $\mathbf{e}^{(\Omega)}_{ij}$, which also depends on the system orientation Ω, and the dipolar coupling constant

$$b_{ij} = -\frac{\mu_0 \gamma^2 \hbar}{4\pi r^3_{ij}}, \tag{S.44}$$

which depends on the distance r_{ij} between the spins.

In typical high-field NMR experiments, the energy scale ω_0 of the global Zeeman field is much larger than the other parts of the Hamiltonian. It is common practice, therefore, to perform the secular approximation after moving the frame rotating with H_0 to get an effective Hamiltonian

$$\tilde{H} = \omega_0 \sum_i \omega_i \left(\mathbf{e}_{\mathrm{cs}}^{(\Omega)} \cdot \mathbf{e}_z\right) S_i^z + \sum_{i,j} d_{ij} \left(2\hat{S}_i^z \hat{S}_j^z - \frac{1}{2}\left(\hat{S}_i^+ \hat{S}_j^- + \hat{S}_i^- \hat{S}_j^+\right)\right) \tag{S.45}$$

The secular dipolar coupling constant is

$$d_{ij} = -\frac{\mu_0 \gamma^2 \hbar}{4\pi r_{ij}^3} \frac{1}{2}\left(3\cos^2\Theta_{ij} - 1\right) \tag{S.46}$$

where Θ_{ij} is the angle between the dipole vector between the spins and the z-axis. Magic angle spinning of the sample at frequency ω_r around an axis at a polar angle θ_r with respect to the z-axis can be included by rewriting the Hamiltonian as [42]

$$\begin{aligned}\tilde{H}_{\mathrm{MAS}} &= \omega_0 \sum_i \left(1 - \delta_i^{\mathrm{iso}}\right) S_i^z \\ &+ \omega_0 \sum_{n=-2:2} d_{n,0}^{(2)}(\theta_r)\, e^{in\omega_r t} \sum_i \delta_i^{\mathrm{aniso}} \tilde{g}_{n,i} S_i^z \\ &+ \sum_{n=-2:2} d_{n,0}^{(2)}(\theta_r)\, e^{in\omega_r t} \sum_{i,j} b_{ij} \tilde{G}_{n,ij} \left(2\hat{S}_i^z \hat{S}_j^z - \frac{1}{2}\left(\hat{S}_i^+ \hat{S}_j^- + \hat{S}_i^- \hat{S}_j^+\right)\right)\end{aligned} \tag{S.47}$$

where we have defined the quantities

$$\delta_i^{\mathrm{iso}} = \frac{1}{3}\left(\delta_i^{XX} + \delta_i^{YY} + \delta_i^{ZZ}\right) \tag{S.48}$$

$$\delta_i^{\mathrm{aniso}} = \delta_i^{ZZ} - \delta_i^{\mathrm{iso}} \tag{S.49}$$

$$\eta_i = \frac{\delta_i^{YY} - \delta_i^{XX}}{\delta_i^{\mathrm{aniso}}} \tag{S.50}$$

$$\tilde{g}_n = \sqrt{\frac{3}{2}} D_{0,n}^{(2)}(\alpha_i, \beta_i, \gamma_i) - \frac{1}{2}\eta_i \left(D_{2,n}^{(2)}(\alpha_i, \beta_i, \gamma_i) + D_{-2,n}^{(2)}(\alpha_i, \beta_i, \gamma_i)\right) \tag{S.51}$$

$$\tilde{G}_{n,ij} = D_{0,n}^{(2)}(0, \beta_{ij}, \gamma_{ij}). \tag{S.52}$$

The geometric factors $d_{n,0}^{(2)}$, $D_{0,n}^{(2)}$, and $D_{\pm 2,n}^{(2)}$ depend on rotor angle θ_r, the Euler angles $(\alpha_i, \beta_i, \gamma_i)$ relating the principal axis frame of each spin to the rotor frame, and the Euler angles $(0, \beta_{ij}, \gamma_{ij})$ relating the dipole vector between spins i and j to the rotor frame.

In solid-state NMR experiments, a sample consists of an ensemble of systems, each with a different orientation. The orientations, Ω, takes a uniformly random but static value during the experiment. We can therefore simulate solid-state NMR and experiments by computing the spectrum for an ensemble of Hamiltonians, Eq. (S.45) or Eq. (S.47), each corresponding to a different orientation Ω_k. Performing the ensemble average over orientations, known as the powder average, is onerous for classical computers as we must do several parallel simulations of instances of the dynamics corresponding to fixed orientations, each of which can be time-consuming for large systems. In the main text, we discuss how a powder-averaged simulation performed on a quantum device has roughly the same resource cost for such systems as the simulation of a single instance of the dynamics.

REFERENCES AND NOTES

1. M. H. Levitt, in *Spin Dynamics: Basics of Nuclear Magnetic Resonance* (John Wiley & Sons Ltd., 2008), pp. 60–61.

2. B. Reif, S. E. Ashbrook, L. Emsley, M. Hong, Solid-state NMR spectroscopy. *Nat. Rev. Methods Primers* **1**, 2 (2021).

3. K. Chen, A practical review of NMR lineshapes for spin-1/2 and quadrupolar nuclei in disordered materials. *Int. J. Mol. Sci.* **21**, 5666 (2020).

4. L. J. Edwards, D. Savostyanov, Z. Welderufael, D. Lee, I. Kuprov, Quantum mechanical NMR simulation algorithm for protein-size spin systems. *J. Magn. Reson.* **243**, 107–113 (2014).

5. T. Theis, P. Ganssle, G. Kervern, S. Knappe, J. Kitching, M. P. Ledbetter, D. Budker, A. Pines, Parahydrogen-enhanced zero-field nuclear magnetic resonance. *Nat. Phys.* **7**, 571–575 (2011).

6. D. A. Barskiy, M. C. D. Tayler, I. Marco-Rius, J. Kurhanewicz, D. B. Vigneron, S. Cikrikci, A. Aydogdu, M. Reh, A. N. Pravdivtsev, J.-B. Hövener, J. W. Blanchard, T. Wu, D. Budker, A. Pines, Zero-field nuclear magnetic resonance of chemically exchanging systems. *Nat. Commun.* **10**, 3002 (2019).

7. A. M. Childs, D. Maslov, Y. Nam, N. J. Ross, Y. Su, Toward the first quantum simulation with quantum speedup. *Proc. Natl. Acad. Sci. U.S.A.* **115**, 9456–9461 (2018).

8. D. Sels, H. Dashti, S. Mora, O. Demler, E. Demler, Quantum approximate Bayesian computation for NMR model inference. *Nat. Mach. Intell.* **2**, 396–402 (2020).

9. L. Egan, D. M. Debroy, C. Noel, A. Risinger, D. Zhu, D. Biswas, M. Newman, M. Li, K. R. Brown, M. Cetina, C. Monroe, Fault-tolerant operation of a quantum error-correction code. arXiv:2009.11482 [quant-ph] (24 September 2020).

10. M. P. Ledbetter, T. Theis, J. W. Blanchard, H. Ring, P. Ganssle, S. Appelt, B. Blümich, A. Pines, D. Budker, Near-zero-field nuclear magnetic resonance. *Phys. Rev. Lett.* **107**, 107601 (2011).

11. M. Bostock, D. Nietlispach, Compressed sensing: Reconstruction of non-uniformly sampled multidimensional NMR data *Concepts Magn. Reson. Part A* **46A**, e21438 (2018).

12. E. Younis, K. Sen, K. Yelick, C. Iancu, Qfast: Conating search and numerical optimization for scalable quantum circuit synthesis. arXiv:2103.07093 [quant-ph] (12 March 2021).

13. A. J. Daley, I. Bloch, C. Kokail, S. Flannigan, N. Pearson, M. Troyer, P. Zoller, Practical quantum advantage in quantum simulation. *Nature* **607**, 667–676 (2022).

14. X. Andrade, J. N. Sanders, Application of compressed sensing to the simulation of atomic systems. *Proc. Natl. Acad. Sci. U.S.A.* **109**, 13928–13933 (2012).

15. T. E. O'Brien, L. B. Ioffe, Y. Su, D. Fushman, H. Neven, R. Babbush, and V. Smelyanskiy, Quantum computation of molecular structure using data from challenging-to-classically-simulate nuclear magnetic resonance experiments. arXiv:2109.02163 [quant-ph] (5 September 2021).

16. D. Donoho, Compressed sensing. *IEEE Trans. Inf. Theory* **52**, 1289–1306 (2006).

17. K. Arai, C. Belthangady, H. Zhang, N. Bar-Gill, S. J. De- Vience, P. Cappellaro, A. Yacoby, R. L. Walsworth, Fourier magnetic imaging with nanoscale resolution and compressed sensing speed-up using electronic spins in diamond. *Nat. Nanotechnol.* **10**, 859–864 (2015).

18. S. G. Hyberts, K. Takeuchi, G. Wagner, Poisson-gap sampling and forward maximum entropy reconstruction for enhancing the resolution and sensitivity of protein NMR data. *J. Am. Chem. Soc.* **132**, 2145–2147 (2010).

19. J. Choi, A. L. Shaw, I. S. Madjarov, X. Xie, J. P. Covey, J. S. Cotler, D. K. Mark, H.-Y. Huang, A. Kale, H. Pichler, F. G. S. L. Brandão, S. Choi, M. Endres, Emergent randomness

and benchmarking from manybody quantum chaos. arXiv:2103.03535 [quant-ph] (5 March 2021).

20. S. Boixo, S. V. Isakov, V. N. Smelyanskiy, R. Babbush, N. Ding, Z. Jiang, M. J. Bremner, J. M. Martinis, H. Neven, Characterizing quantum supremacy in near-term devices. *Nat. Phys.* **14**, 595–600 (2018).

21. E. Matern, M. Engelhardt, G. Haegele, Solving the $^{31}P\{^{1}H\}$ NMR spectrum of $(Me_3Si)_3P_7$ as $B[AC]_3$ case. *Phosphorus Sulfur Silicon Relat. Elem.* **192**, 727–731 (2017).

22. K. R. Brown, J. Kim, C. Monroe, Co-designing a scalable quantum computer with trapped atomic ions. *npj Quantum Inf.* **2**, 16034 (2016).

23. H. Hogben, M. Krzystyniak, G. Charnock, P. Hore, I. Kuprov, Spinach – A software library for simulation of spin dynamics in large spin systems. *J. Magn. Reson.* **208**, 179–194 (2011).

24. A. M. Childs, Y. Su, M. C. Tran, N. Wiebe, S. Zhu, Theory of trotter error with commutator scaling. *Phys. Rev. X* **11**, 011020 (2021).

25. J. P. Gaebler, T. R. Tan, Y. Lin, Y. Wan, R. Bowler, A. C. Keith, S. Glancy, K. Coakley, E. Knill, D. Leibfried, D. J. Wineland, High-fidelity universal gate set for $^{9}Be^{+}$ ion qubits. *Phys. Rev. Lett.* **117**, 060505 (2016).

26. C. J. Ballance, T. P. Harty, N. M. Linke, M. A. Sepiol, D. M. Lucas, High-fidelity quantum logic gates using trapped-ion hyperfine qubits. *Phys. Rev. Lett.* **117**, 060504 (2016).

27. J. Tindall, M. Fishman, M. Stoudenmire, D. Sels, Efficient tensor network simulation of IBM's kicked ising experiment. arXiv:2306.14887 [quant-ph] (26 June 2023).

28. M. Kühn, S. Zanker, P. Deglmann, M. Marthaler, H. Weib, Accuracy and resource estimations for quantum chemistry on a near-term quantum computer. *J. Chem. Theory Comput.* **15**, 4764–4780 (2019).

29. J. F. Gonthier, M. D. Radin, C. Buda, E. J. Doskocil, C. M. Abuan, J. Romero, Identifying challenges towards practical quantum advantage through resource estimation: The measurement roadblock in the variational quantum eigensolver. arXiv:2012.04001 [quant-ph] (7 December 2020).

30. K. Seetharam, D. Sels, E. Demler, Platform tailored co-design of gate-based quantum simulation. arXiv:2111.00024 [quant-ph] (29 October 2021).

31. R. Ghose, Average Liouvillian theory in nuclear magnetic resonance – Principles, properties, and applications. *Concepts Magn. Reson.* **12**, 152–172 (2000).

32. E. Vlachos, H. Zhang, V. Maurya, J. Marshall, T. Albash, E. M. Levenson-Falk, Master equation emulation and coherence preservation with classical control of a superconducting qubit. *Phys. Rev. A.* **106**, 062620 (2022).

33. M. A. Nielsen, A simple formula for the average gate fidelity of a quantum dynamical operation. *Phys. Rev. A.* **303**, 249–252 (2002).

34. Y. Li, S. C. Benjamin, Efficient variational quantum simulator incorporating active error minimization. *Phys. Rev. X.* **7**, 021050 (2017).

35. D. V. Savostyanov, S. V. Dolgov, J. M. Werner, I. Kuprov, Exact NMR simulation of protein-size spin systems using tensor train formalism. *Phys. Rev. B* **90**, 085139 (2014).

36. J. T. Merrill, K. R. Brown, Progress in compensating pulse sequences for quantum computation, in *Quan-tum Information and Computation for Chemistry* (John Wiley & Sons Ltd., 2014) pp. 241–294.

37. K. Mølmer, A. Sørensen, Multiparticle entanglement of hot trapped ions. *Phys. Rev. Lett.* **82**, 1835 (1999).

38. I. Kuprov, Large-scale NMR simulations in liquid state: A tutorial. *Magn. Reson. Chem.* **56**, 415–437 (2018).

39. S. L. Braunstein, C. M. Caves, R. Jozsa, N. Linden, S. Popescu, R. Schack, Separability of very noisy mixed states and implications for NMR quantum computing. *Phys. Rev. Lett.* **83**, 1054–1057 (1999).

40. N. C. Menicucci C. M. Caves, Local realistic model for the dynamics of bulk-ensemble NMR information processing. *Phys. Rev. Lett.* **88**, 167901 (2002).

41. Y. Zhou, E. M. Stoudenmire, X. Waintal, What limits the simulation of quantum computers?. *Phys. Rev. X* **10**, 041038 (2020).

42. M. Leskes, P. Madhu, S. Vega, Floquet theory in solid-state nuclear magnetic resonance. *Prog. Nucl. Magn. Reson. Spectrosc.* **57**, 345–380 (2010).
